# The MelFo Study UK: Effects of a Reduced-Frequency, Stage-Adjusted Follow-Up Schedule for Cutaneous Melanoma 1B to 2C Patients After 3-Years

**DOI:** 10.1245/s10434-020-08758-2

**Published:** 2020-07-04

**Authors:** Marc D. Moncrieff, Beverly Underwood, Jennifer J. Garioch, Martin Heaton, Nakul Patel, Esther Bastiaannet, Josette E. H. M. Hoekstra-Weebers, Harald J. Hoekstra

**Affiliations:** 1 Department of Plastic and Reconstructive Surgery, Norfolk and Norwich University Hospital, Norwich, UK; 2 Department of Dermatology, Norfolk and Norwich University Hospital, Norwich, UK; 3 Leiden University Medical Center, Leiden, The Netherlands; 4 University Medical Center Groningen, Wenckebach Institute, University of Groningen, Groningen, The Netherlands; 5 Netherlands Comprehensive Cancer Organisation, Groningen, The Netherlands; 6 Department of Surgical Oncology, University Medical Center Groningen, University of Groningen, Groningen, The Netherlands; 7 University of East Anglia, Norwich Research Park, Norwich, Norfolk, NR4 7TJ UK

## Abstract

**Background:**

Evidence-based guidelines for follow-up treatment of American Joint Committee on Cancer (AJCC) stages 1B to 2C melanoma patients are lacking. The MELanoma FOllow-up study is an international phase 3 randomized trial, and the 3-year interim data were recently reported from the Netherlands. The study was undertaken concurrently with a British cohort for comparison and validation of the Dutch study.

**Methods:**

The study enrolled and stratified 207 patients by AJCC stage. The conventional schedule group (CSG; *n* = 103) cohort was reviewed as per UK guidelines. The experimental schedule group (ESG; *n* = 104) cohort was reviewed in a reduced-frequency nurse-led, consultant-supervised clinic. Quality of life (QoL) was measured at baseline (T1), a 1 year (T2), and at 3 years (T3) using the State-Trait Anxiety Inventory, the Cancer Worry Scale, the Impact-of-Event Scale, and the Mental and Physical Component scales (PCS/MCS) of the RAND-36.

**Results:**

Of the 207 QoL questionnaires, 170 (82.1%) were completed at T3. Both cohorts expressed high satisfaction (> 93%) with their regimens. At T3, no significant group effect was found on any patient-reported outcome measures scores, indicating no QoL difference between the follow-up protocols. Recurrence had developed in 33 patients Conventional follow-up (CFU), 16 [15.5%]; Experimental follow-up (EFU), 17 [16.3%]. Self-examination was the method of detection for 12 ESG patients (70.6%) and 11 CSG patients (68.8%). The melanoma-specific survival was identical.

**Conclusion:**

The UK 3-year data were consistent with the previous Dutch report. The reduced follow-up strategy was shown to be safe, with significant resource usage benefits for national cancer services. Patient anxiety levels were not increased by a less-intensive follow-up regimen, and acceptance was high. The study data indicate that patient self-examination is very effective for recurrence detection.

Primary cutaneous melanoma is the fifth most common cancer in the UK, accounting for 4% of all new cancer cases, and since the early 1990s, melanoma incidence rates have more than doubled (128%).^1^ The incidence rates for melanoma skin cancer are projected to rise by 7% in the UK between 2014 and 2035, to 32 cases per 100,000 individuals by 2035.

Melanoma disproportionately affects a younger demographic relative to other solid human cancers, with a melanoma diagnosis for nearly half of the patients before their 65th birthday. Furthermore, the prognosis for melanoma generally is very good^2^ (overall 10-year survival rate of 90%). It is estimated that more than 150,000 people in the UK currently are living with the diagnosis of melanoma. Therefore, long-term follow-up arrangements and patient education for early detection has become a key survivorship issue.

The routine use of sentinel lymph node biopsy (SLNB) for accurate staging of melanoma patients has been incorporated into most international melanoma guidelines. Although the purpose of SLNB may have subtly but firmly shifted from identifying high-risk patients requiring a completion lymph node dissection to stratifying high-risk patients for adjuvant systemic therapy,^3^^–^5 the initial outcome remains the same for the majority of patients in that no further treatment is indicated because their SLNB shows no evidence of melanoma metastasis. However, these patients still require follow-up evaluation because the risk of locoregional or distant spread remains a possibility.

The main aims of follow-up programs for melanoma patients are thought to be early detection of recurrences and prompt recognition of subsequent primary melanoma. Other aims are patient reassurance and evaluation of the surgical treatment outcome. Several groups have attempted to determine the most effective follow-up schedule by testing their current follow-up schedule or by estimating a new follow-up schedule on the basis of retrospective data.^6^^–^9

Most proposed follow-up schedules are based on the premise that the annual risk of recurrence increases with advancing American Joint Committee on Cancer (AJCC) stage.^10^^–^12 Almost 90% of recurrences are experienced in the first 3 years after the primary diagnosis for intermediate and thick melanomas,^7^,^9^,^13^^–^15 and the risk of recurrence after 10 years of follow-up evaluation is low (2.4%).^16^ For thin melanomas, the risk of recurrence is very low in general, although the patients who do go on to experience a recurrence generally present after a significant delay.^17^ Accordingly, national guidelines committees find it challenging to devise simple follow-up schedules for melanoma patients.

The MELanoma FOllow-up (MelFO) study is an international phase 3 randomized controlled trial (RCT), and the 3-year interim data were reported recently from the Netherlands.^18^ This clinical trial aimed to provide an evidence basis for the follow-up evaluation of cutaneous melanoma patients with no evidence of sentinel node metastasis. The primary end points of this trial are related to quality of life (QoL), cancer worry, and stress-related symptoms. The trial was undertaken concurrently in the UK to compare and validate the findings of a Dutch study analyzing the primary end points in an English-speaking cohort, with the additional predetermined intention of ultimately combining the international data set to assess the secondary end points of recurrence rates and survival (patient safety).

The trial hypothesis was that QoL does not decrease with a reduced-intensity AJCC stage-adjusted follow-up regimen relative to the UK National Institute for Health and Care Excellence (NICE)-recommended follow-up regimen for AJCC stages 1B to 2C melanoma patients staged with SLNB. The predetermined interim analysis point was 3 years to ensure patient compliance with the protocol and patient safety in terms of recurrence rates. Accordingly, we report the results of a planned 3-year UK interim analysis of the data.

## Methods

### Study Design

The detailed methods of this multicenter, randomized clinical trial (NCT0108004), initiated by the Department of Surgical Oncology at the University Medical Center of Groningen (UMCG), have been described previously.^19^ The participants were randomized into two groups: one group who followed the conventional schedule recommended in the UK NICE melanoma guidelines^20^ and one group whose follow-up evaluation was an AJCC stage-adjusted reduced schedule (Table 1).

**Table 1:** Frequency of follow-up visits for the conventional follow-up schedule as recommended by the UK NICE Melanoma Guideline,^20^ and a reduced and stage-adjusted experimental follow-up schedule

Conventional follow-up schedule							Difference at 5 years (*n* visits)	Experimental follow-up schedule						
Years^a^	1	2	3	4	5	6–10		Years^a^	1	2	3	4	5	6–10
AJCC stage	Visits per year							AJCC stage	Visits per year					
1B	4	4	4	2	2	0	11	IB	1	1	1	1	1	1
2A	4	4	4	2	2	1	9	IIA	2	2	1	1	1	1
2B	4	4	4	2	2	1	6	IIB	3	3	2	1	1	1
2C	4	4	4	2	2	1	6	IIC	3	3	2	1	1	1

*AJCC* American Joint Committee on Cancer

^a^Year after surgery for primary melanoma, including staging with sentinel node biopsy

The primary end point for this study was patient QoL. The secondary end points were recurrence rates, site of recurrence, and method of detection, in addition to standard outcomes data such disease-specific and overall survival findings. The study was approved by the Cambridgeshire research ethics committee service (Rec Ref: 10/H0306/18; IRAS number: 43852).

### Patients and Procedure

The inclusion criteria specified SLNB-negative melanoma patients with AJCC stages 1B to 2C disease who had undergone surgery with curative intent between 2010 and 2015. The study excluded patients younger than 18 years or older than 85 years, those not able to speak English and/or unable to complete the questionnaires, and those who had another malignancy.

After giving informed consent, eligible patients were randomized into the conventional group (CSG) or the experimental schedule group (ESG) in a 1:1 manner and stratified according to AJCC stage. The Netherlands Comprehensive Cancer Organization (IKNL) was the coordinating clinical trial center for this study and performed the randomization of the UK patients.

The patients completed questionnaires at study entry shortly after diagnosis (T1), after 1 year (T2), and 3 years later (T3). Patients were excluded from T2 or T3 if they withdrew consent or died. Clinicians provided follow-up information on the disease status and overall performance status of all the patients during the 3 years of the study or until the patients experienced a recurrence, a second primary tumor, or death.

The current study focused on comparisons between the T1, T2, and T3 time points, with QoL as a primary end point. Recurrence detection rates, clinical outcomes and patient satisfaction rates, and schedule compliance were secondary end points.

### QOL/PROMs Instruments

At T1, the patients answered questions on gender, age, level of education, relationship status, daily activities, and comorbidities. At T1 and T3, they answered questions on schedule satisfaction, frequency of self-inspection, and number of melanoma-related general practitioner/primary care physician (GP) visits. The treating clinicians gave diagnostic information (primary melanoma site, Breslow thickness, ulceration, AJCC classification) and follow-up information (date of every outpatient visit, date and location of recurrence, date and cause of death). The patients completed the following patient-reported outcome measures (PROMs) at T1, T2, and T3:

1. The State-Trait Anxiety Inventory-state version (STAI-s), a 20-item questionnaire measuring the transitory emotional condition of stress or tension perceived by the patient. Items are scored on a 4-point scale ranging from 1 (not at all) to 4 (very much) (range, 20–80).^21^
2. The 3-item Cancer Worry Scale (CWS) measuring concerns about cancer developing again and the impact on daily activities.^22^^–^24 Higher scores mean more worries (range, 3–12).
3. The 15-item Impact-of-Event Scale (IES) evaluating the extent to which patients experience life hazards, in this case having a melanoma, in terms of avoidance and intrusion.^25^, ^26^ A higher score (range, 0–75) corresponds to a higher level of stress response symptoms.
4. The RAND-36, a 36-item health-related QoL questionnaire, of which the mental component score (MCS) and the physical component summary scores (PCS) were used. The summary scores are standardized with a mean of 50 and a standard deviation of 10.^27^

### Statistical Analysis

Statistical analyses were performed using IBM SPSS statistics version 22 (SPSS Inc; Chicago, IL, USA) and STATA v.12 (StataCorp, College Station, TX, USA). The sample size and power analyses have been described previously.^19^ Patient characteristics were described, and comparisons between study groups were performed using independent *t* tests, the Mann–Whitney *U* test, Chi square tests, or Fisher’s exact tests, as appropriate. To examine differences between groups and time differences in PROMs, *t* tests and paired *t* tests were performed. When a difference was found to be statistically significant, effect sizes were computed to examine clinical relevance. Clinicians consider effect size values of 0.5 or higher to be large, those between 0.3 and 0.5 to be moderate, and those lower than 0.3 to be small.^28^ Kaplan–Meier log-rank tests were performed to compare groups in terms of recurrence-free survival and disease-specific survival. In all the statistical analyses, *p* values lower than 0.05 were considered statistically significant.

## Results

**Fig. 1:**
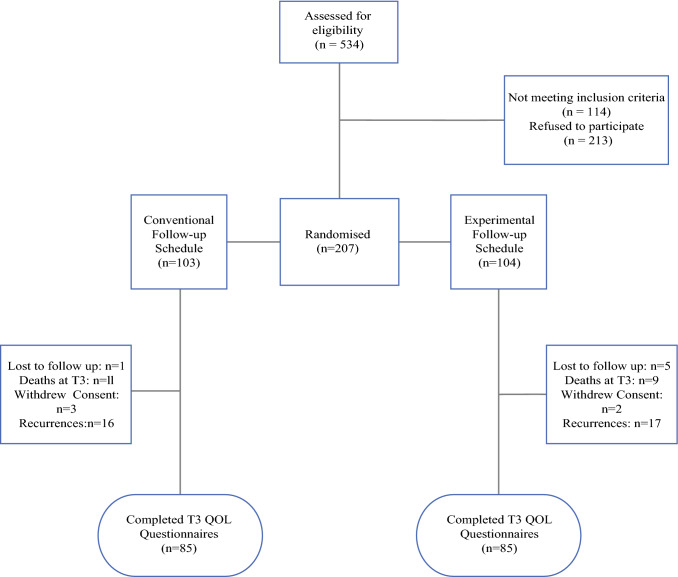
Consort diagram for Mel-FO

Enrolment of the patients and their outcomes are summarized in the Consolidated Standards of Reporting Trials (CONSORT) diagram (Fig. 1). In summary, 534 patients were assessed for eligibility, and 114 did not meet the inclusion criteria. Ultimately, 207 of the 420 eligible patients (response rate, 49.2%) were enrolled in the study (99 women and 108 men; median age, 62 years; interquartile range [IQR], 52–69 years) and stratified by AJCC stage. The conventional schedule group (CSG; *n* = 103) cohort was reviewed clinically as per national guidelines.^20^ The experimental schedule group (ESG; *n* = 104) cohort was reviewed according to a reduced-frequency schedule (Table 1). For both cohorts, follow-up evaluation was performed in a cancer nurse specialist (CNS)-led/consultant-supervised melanoma clinic in the combined skin cancer outpatient department using structured patient education for self-examination techniques at the time of enrolment. The patient education component for self-examination was reinforced by the CNS at each scheduled visit.

Table 2 describes the distribution of the patients and their tumors between the two experimental cohorts. The two cohorts were well-matched for age, education/marital/employment statuses, and tumor stage. Gender was an exception, with significantly more women in the ESG cohort (55.8% vs. 39.8%; *p* = 0.02).

**Table 2:** Patient and melanoma characteristics at randomization T1 for the study groups

Characteristics		Total (*n* = 207) *n* (%)	Conventional schedule (*n* = 103) *n* (%)	Experimental schedule (*n* = 104) *n* (%)	*p* Value
Sex	Female	99 (47.8)	41 (39.8)	58 (55.8)	0.02
	Male	108 (52.2)	62 (60.2)	46 (44.2)	
Age (years)	≤ 55	68 (32.9)	36 (35.0)	32 (30.8)	0.46
	56–64	62 (29.9)	33 (32.0)	29 (27.9)	
	65+	77 (37.2)	34 (33.0)	43 (41.3)	
Level of education	Primary school	2 (1.0)	2 (1.9)	0 (0.0)	0.34
	Secondary/high	133 (64.3)	70 (68.0)	63 (60.6)	
	Diploma	27 (13.0)	11 (10.7)	16 (15.4)	
	University	42 (20.3)	18 (17.5)	24 (23.1)	
	Unknown	3 (1.4)	2 (1.9)	1 (1.0)	
Relationship status	Without partner	34 (16.4)	13 (12.6)	21 (20.2)	0.21
	With partner	172 (83.1)	89 (86.4)	83 (79.8)	
	Unknown	1 (0.5)	1 (1.0)	0 (0.0)	
Daily activities	Employed	95 (45.9)	52 (50.5)	43 (41.3)	0.41
	Not employed	110 (53.1)	50 (48.5)	60 (57.7)	
	Unknown	2 (1.0)	1 (1.0)	1 (1.0)	
Presence of comorbidities	No	119 (57.5)	60 (58.3)	59 (56.7)	0.37
	Yes	86 (41.5)	43 (41.7)	43 (41.4)	
	Unknown	2 (1.0)	0 (0.0)	2 (1.9)	
Primary melanoma	Head and neck	34 (16.4)	15 (14.6)	19 (18.3)	0.22
	Trunk	82 (39.6)	47 (45.6)	35 (33.7)	
	Lower extremity	45 (21.8)	23 (22.3)	22 (21.1)	
	Upper extremity	46 (22.2)	18 (17.5)	28 (26.9)	
Breslow thickness	< 1.00	47 (22.7)	21 (20.4)	26 (25.0)	0.47
	1.00–2.00	103 (49.8)	55 (53.4)	48 (46.2)	
	2.01–4.00	44 (21.2)	19 (18.4)	25 (24.0)	
	> 4.00	13 (6.3)	8 (7.8)	5 (4.8)	
Ulceration	No	166 (80.2)	85 (82.5)	81 (77.9)	0.40
	Yes	41 (19.8)	18 (17.5)	23 (22.1)	
AJCC classification	1B	136 (65.7)	68 (66.0)	68 (65.4)	0.97
	2A	33 (15.9)	16 (15.5)	17 (16.3)	
	2B	33 (15.9)	16 (15.5)	17 (16.4)	
	2C	5 (2.4)	3 (2.9)	2 (1.9)	

*AJCC* American Joint Committee on Cancer

At the T3 time point, 154 (88.2%) of 170 of patients completed the follow-up questionnaires. Table 3 demonstrates that after 3 years, no significant group difference was found in terms of patient satisfaction with the follow-up schedule, with both groups expressing satisfaction at a rate higher than 93%. Nearly all the patients in both groups reported examining their skin and lymph node fields, and both groups were performing this with a similar frequency.

**Table 3:** Follow-up related questions at 3 years (T4)

Follow-up related questions at 3 years	Total (*n* = 154) *n* (%)	Conventional schedule (*n* = 79) *n* (%)	Experimental schedule (*n* = 75) *n* (%)	*p* Value
*Schedule satisfaction*				
Yes	145 (94.2)	74 (93.7)	71 (94.6)	0.92
No	5 (3.2)	3 (3.8)	2 (2.7)	
Missing	4 (2.6)	2 (2.5)	2 (2.7)	
*Reason dissatisfaction*				
Wants more visits	2 (1.3)	1 (1.3)	1 (1.3)	0.62
Wants fewer visits	1 (0.6)	1 (1.3)	0 (0.0)	
*Melanoma-related GP visits (last 6* *months)*				
None	65 (42.2)	29 (36.7)	36 (48.0)	0.31
Every week	6 (3.9)	3 (3.8)	3 (4.0)	
Once every month	0 (0.0)	0 (0.0)	0 (0.0)	
Every 3 months	1 (0.6)	0 (0.0)	1 (1.3)	
Less than every 3 months	0 (0.0)	0 (0.0)	0 (0.0)	
Never	1 (0.6)	0 (0.0)	1 (1.3)	
Unknown	81 (52.6)	47 (59.5)	34 (45.3)	
*Frequency of skin self-inspection*				
Every week	54 (35.1)	26 (32.9)	28 (37.3)	0.72
Every month	74 (48.0)	42 (53.1)	32 (42.7)	
Once every 3 months	15 (9.7)	7 (8.9)	8 (10.7)	
Less than every 3 months	7 (4.6)	3 (3.8)	4 (5.3)	
Never	1 (0.7)	0 (0.0)	1 (1.3)	
Unknown	3 (1.9)	1 (1.3)	2 (2.7)	
*Frequency of lymph node self-inspection*				
Every week	45 (29.2)	20 (25.3)	25 (33.3)	0.18
Every month	78 (50.7)	44 (55.7)	34 (45.3)	
Once every 3 months	16 (10.4)	8 (10.1)	8 (10.7)	
Less than every 3 months	9 (5.8)	2 (2.5)	7 (9.3)	
Never	2 (1.3)	2 (2.5)	0 (0.0)	
Unknown	4 (2.6)	3 (3.8)	1 (1.3)	

*GP* melanoma-related general practitioner/primary care physician

Table 4 shows that the overall compliance with the follow-up schedules was high at the T2 and T3 time points (68.5% and 66.5%, respectively). At T2, no significant group difference in overall compliance with the follow-up schedule was observed (ESG, 69.9% vs. CSG, 67%). However, significantly more scheduled visits were made by the ESG patients (25.2%) than by the CSG patients (11%), and significantly more scheduled visits were unattended by the CSG patients (22%) than by the ESG patients (4.9%). This trend was statistically significant (Pearson Chi square test [*df* = 2], *p* < 0.0001; test for trend [*df* = 1], *p* = 0.0006). At T3, no significant group effect was observed with the schedule compliance rate (CSG, 71.8% vs. ESG, 61.2%). Furthermore, no significant differences between the groups were detected in the number of unscheduled or missed follow-up appointments. Of the 31 patients who did access an additional clinic in the 12 months before the T3 time point, 38 (81.6%) did this only once. At the same time, only 4.6% indicated that they had visited their GP in the preceding 6 months. The majority of the extra visits were for other suspicious lesions, which eventually were diagnosed as benign lesions or non-melanoma skin cancers (16/38 patients, 42.1%). Of the 38 patients, 12 (31.6%) chose to rearrange their appointments to suit their lifestyle (including 1 pregnancy) rather than for any specific cancer concerns. The remaining patients (26.3%) were concerned about signs or symptoms related to cancer recurrence.

**Table 4:** Follow-up schedule compliancy and detection of recurrences

	Total (*n* = 207) *n* (%)	Conventional schedule (*n* = 103) *n* (%)	Experimental schedule (*n* = 104) *n* (%)	*p* Value
*Schedule compliance (0–1* *years)*				
Missed visits	27 (13.3)	22(22)	5 (4.9)	For trend
On schedule	139 (68.5)	67(67)	72 (69.9)	0.0001
Extra visits	37 (18.2)	11 (11)	26 (25.2)	
Off trial	4	3	1	
*Schedule compliance (2–3* *years)*				
Missed visits	19 (11.2)	9 (10.6)	10 (11.8)	NS
On schedule	113 (66.5)	61 (71.8)	52 (61.2)	
Extra visits	38 (22.3)	15 (17.6)	23 (27.1)	
Off trial	37	18	19	
*Site of initial recurrence*				
Local/in-transit	6 (18.2)	0	6 (23.5)	
Regional nodes	8 (24.2)	4 (25.0)	4 (23.5)	
Distant	10 (30.3)	7 (43.8)	3 (17.6)	
Multiple sites	5 (15.2)	3 (18.8)	2 (11.8)	
Second melanoma	4 (12.1)	2 (12.5)	2 (11.8)	
Second malignancy	3	3	0	
*Detection*				
Patient	23 (69.7)	11 (68.8)	12 (70.6)	NS
Relative	1 (3)	1 (6.3)	0	
Clinician	9 (27.3)	4 (25)	5 (29.4)	
*AJCC stage (8th ed)*				
1A	2 (6.1)	1 (6.3)	1 (5.9)	NS
1B	9 (27.3)	3 (18.8)	6 (35.3)	
2A	9 (27.3)	4 (25)	5 (29.4)	
2B	10 (30.3)	6 (37.5)	4 (23.5)	
2C	3 (9.1)	2 (12.5)	1 (5.9)	

*NS* not significant; *AJCC* American Joint Committee on Cancer

### Patient-Reported Outcome Measures

The QoL questionnaires were completed by 184 patients at T2 (94 ECG and 90 CSG patients) and 170 patients at T3 (85 ESG and 85 CSG patients). Table 5 describes the QoL/PROMs data. At T1, the two groups did not differ significantly in terms of QoL/PROMs measurements, except for the CWS. The CSG cohort had a significantly higher CWS than the ESG cohort (8.4 vs. 7.4; *p* = 0.02). At T2, no significant group effect on the IES, CWS, STAI, or RAND-36 scores was found, indicating no difference in QoL between the follow-up protocols. Comparison of the T1 and T2 QoL data showed a significant improvement in the CWS and IES for the CSG cohort (*p* < 0.001 and *p* = 0.006, respectively), indicating that the patients were experiencing fewer stress response symptoms and less worry related to their cancer in the CSG cohort after 1 year than shortly after diagnosis.

**Table 5:** Quality of life and patient-reported outcome measures at diagnosis, after 1 year (*n* = 184), and after 3 years (*n* = 170)

Questionnaire	Study group	Mean T1 at randomization*	Mean T2 at 1 year	*p* value for study group		*p* value for time	Mean T3 at 3 years	*p* value for study group	*p* value for time
				T1	T2		T3		
State-Trait Anxiety Inventory	Conv	32.8 ± 17.8	31.6 ± 10.9	0.62	0.84	0.53 (Conv)	33.5 ± 15.9	0.65	0.82 (Conv)
	Expl	34.2 ± 19.7	32.0 ± 10.9			0.28 (Exp)	35.0 ± 22.9		0.79 (Exp)
Cancer Worry Scale	Conv	8.4 ± 3.2	7.1 ± 2.5	0.02 ES = 0.32	0.74	< 0.001 (Conv) ES = 0.46	6.8 ± 2.0	0.32	< 0.001 (Conv) ES = 0.71
	Exp	7.4 ± 3.1	6.9 ± 2.5			0.17 (Exp)	6.5 ± 2.0		0.009 (Exp) ES = 0.4
Impact-of-Event Scale	Conv	26.6 ± 10.8	22.8 ± 9.1	0.50	0.75	0.006 (Conv) ES = 0.47	19.5 ± 7.0	0.47	< 0.001 (Conv) ES = 0.88
	Exp	25.6 ± 9.9	22.9 ± 9.0			0.007 (Exp) ES = 0.29	20.6 ± 8.1		< 0.001 (Exp) ES = 0.57
RAND-36 mental component	Conv	50.9 ± 9.7	53.0 ± 7.8	0.86	0.31	0.02 (Conv)	53.0 ± 9.3	0.99	0.15 (Conv)
	Exp	50.7 ± 10.8	51.6 ± 9.6			0.38 (Exp)	53.0 ± 8.4		0.27 (Exp)
RAND-36 physical component	Conv	49.1 ± 9.6	50.4 ± 10.6	0.97	0.35	0.23 (Conv)	50.9 ± 10.3	0.77	0.11 (Conv)
	Expl	49.0 ± 10.2	49.0 ± 11.1			0.93 (Exp)	50.4 ± 9.1		0.15 (Exp)

*Conv* conventional, *Exp* experimental, *ES* effect size (Cohen’s *d*)

The ESG cohort showed no difference in QoL scores between T1 and T3, except for the IES (*p* = 0.007), indicating that the ESG cohort was experiencing fewer stress response symptoms during the first year. At T3, no significant group effect on the IES, CWS, STAI, or RAND-36 scores was found, indicating no difference in QoL between the follow-up protocols. Comparison of the T1 and T3 data showed that both the ESG and CSG cohorts were experiencing significantly fewer stress response symptoms and less cancer worry after 3 years, indicating that the sustained improvement in QoL observed in the two groups at T2 had been maintained at T3. Effect size calculations (Cohen’s d) indicated that the clinical importance of the observed statistically significant between-group difference in CWS at T1 was small (effect size, 0.32). The effect size calculated on statistically significant time differences ranged between small (effect size of 0.29 for the difference between T1 and T2 in IES in the ESG cohort) and clinically important (ES of 0.88 for the difference between T1 and T3 in IES in the CSG cohort) and showed that improvements in CWS and stress response symptoms measurements became greater as time passed, particularly in the ESG cohort.

### Melanoma Recurrences and Deaths During the 3-Year Follow-Up Period

At T3, 11 patients in the ESG cohort and 9 patients in the CSG cohort had died. The majority of the deaths were melanoma-specific (7/9 [77.8%] in the CSG cohort and 8/11 [72.7%] in the ESG cohort), and the melanoma-specific mortality did not differ between the two groups. Similarly, the two groups did not differ significantly in overall detected recurrence rates or progression-free survival (ESG, 17/104 [16.3%] vs. CSG, 16/103 [15.1%]). In both cohorts, more than two thirds of the recurrences and second melanomas were detected by the patient initially (Table 4). The CSG cohort had a greater proportion of distant recurrences than the ESG cohort, which had a greater proportion of locoregional recurrences at the initial sites of recurrence, although this difference was not statistically significant (*p* = 0.095, Fisher’s exact test). No AJCC initial stage-specific bias was observed between the two cohorts in terms of recurrence rates (Chi square test for trend, *p* = 0.254).

## Discussion

This study showed that 3 years after staging with a negative SLNB, the AJCC stages 1B to 2C cutaneous melanoma patients assigned to the reduced, stage-adjusted follow-up schedule (ESG) did not differ in levels of anxiety, cancer worry, or mental health-related QoL from the patients assigned to the follow-up schedule as currently advised in the UK NICE melanoma guideline.^20^ Moreover, the ESG patients reported significantly lower levels of cancer worry than the CSG patients from the earliest point of the study (at inclusion). This study demonstrated that the reduced follow-up schedule was safe, with no difference in progression-free or disease-specific survival. These results support our hypotheses of no differences in PROMs, recurrences, or deaths between the study groups.

These results mirror the findings of the Dutch group, which recently reported very similar results in 2019.^18^ As in the Dutch study, our data indicated that the large majority of patients were satisfied with their follow-up regimen regardless of the schedule. The data suggest that patients undergo a period of adjustment during the first 12 months of the follow-up period after treatment before they settle into the follow-up routine. Although the patients in this study were overwhelmingly satisfied with their follow-up regimens, in the early months, they were more inclined to seek extra appointments in the reduced-frequency ESG schedule (mostly to discuss wound-healing issues or to repeat the education session rather than for true cancer concerns). The others were more likely to miss their appointments in the higher-frequency CSG schedule, mostly due to clashes with other lifestyle events, such as vacations or work. During this period, both sets of patients demonstrated significantly reduced levels of worry and fewer cancer stress response symptoms after the initial 12 months of follow-up evaluation, which then persisted through to 3 years.

This study demonstrated significantly less cancer worry in the ESG cohort than in the CSG cohort at study inclusion, although the effect size calculation suggested that the difference was small and not clinically relevant, and therefore unlikely to have biased the results. Previous studies have suggested that 50% of patients report high levels of anxiety before and during outpatient clinic visits.^29^ Our data suggest that the stress response and worry symptoms decrease over time from diagnosis regardless of the follow-up schedule, particularly where no recurrence is detected. Effect size calculations showed that the decreases in clinical importance ranged from small to high in both groups.

Unlike the Dutch study, the two groups in the current study did not differ significantly in the proportion of patients paying extra visits to the specialist clinic. More than 80% of the patients who did access an additional clinic had visited the clinic only once, whereas very few patients (< 5%) had visited their GP in the preceding 6 months. Our data suggest that the reason for these extra visits may have been increased awareness of suspicious lesions, possibly resulting from effective education on self-inspection.^9^,^13^^–^15,29^–^33.

The current 3-year results show that the number of recurrences and second primary melanomas as well as the time until detection for the patients with AJCC stages 1B to 2C disease was independent of the assigned follow-up schedule, which is consistent with the Dutch 3-year MelFO results.^18^ Almost half of recurrences (16/33) were detected within the first 12 months. Consistent with previous literature, this shows that the highest proportion of melanoma recurrences and second primaries is detected during the first year of follow-up evaluation and that the proportion declines in the following years.^7^,^13^^–^15

This study also showed that the patients were most likely to detect their recurrences first, with 75.8% of all recurrences detected this way. This is consistent with observations of previously published studies.^14^,^15^,^29^ In this study, we observed a trend toward the earlier AJCC stage 3 recurrences being detected more frequently in the ESG cohort, which suggests that patient education for self-examination is very effective. Crucially, the study found no evidence of diagnostic delay, with the recurrence-free survival intervals identical in both cohorts. Overall, the 3-year recurrence rate in the current study was 15.9%, which is comparable with the Dutch MelFO cohort rate of 13.4% and that of previously published literature.^13^

One limitation of this study was that the physical examination-based follow-up regimen may be deemed less than adequate for the care of future melanoma patients. Since the start of this trial, effective systemic therapy has become routinely available to patients with advanced melanoma.^34^^–^36 Our protocol for this trial did not mandate any radiologic surveillance of either cohort because there was no convincing evidence showing it to be effective and no consensus on the topic had been reached. However, future follow-up regimens for patients receiving adjuvant systemic therapy likely will include initial radiologic screening and serial surveillance scans to detect asymptomatic stage 4 recurrences. Regardless, the majority of melanoma patients remain AJCC stage 1B/2A (84.1% of the current cohort) after their initial surgical treatment and staging with SLNB, and these patients would be unlikely to require any further systemic treatment. For this group, the reduced-intensity follow-up schedule still would be valid, and it was encouraging that 75% of all recurrences were detected by the patients themselves.

The current study had several limitations. First, due to the pragmatic design and the open-access clinic policy mandated by both the trial protocol and the ethics committee, one third of the patients did not strictly adhere to the follow-up protocol. Similarly, the dropout rates for the Qol/PROMs measurements were 11% at T2 and 18% at T3. This was higher than the predicted 10% rate envisaged in the trial design.

Second, the power analysis showed that 89 patients per group were needed. We started with 103 patients in the CGS cohort and 104 patients in the ESG cohort, but due to the dropout during 3 years, the number of patients analyzed at T3 was slightly lower than envisaged (85 per group). However, no differences in sociodemographic or illness-related variables were found between the participants in the two study groups at T1.

Third, due to the low event rate in both cohorts, some of the analyses performed, particularly those involving clinical disease outcomes, should be interpreted carefully. Finally, 213 patients of the eligible cohort declined to enroll into this study. For the majority of these patients (> 85%), the distance required for travel to the trial center for follow-up evaluation was the reason given for refusing to participate (data not shown).

## Conclusion

The interim results of this phase 3 RCT at 3 years, undertaken in a native English-speaking cohort, appear to have validated the findings of our Dutch colleagues who undertook this study in the Netherlands using the same protocol.^18^ The UK MelFO study seems to support the notion that a reduced stage-adjusted follow-up schedule is an appropriate and safe approach for AJCC stages 1B to 2C melanoma patients after staging with sentinel biopsy in terms of QoL, patient satisfaction, and disease safety at 3 years. We anticipate reporting the final outcome of the study at the end of 2020, with the data from the Netherlands and the UK combined to ensure adequate power to detect any difference in recurrence rates, thereby dispelling any lingering concerns about patient safety with a reduced follow-up regimen.
